# Iron Status Affects the Zinc Accumulation in the Biomass Plant Szarvasi-1

**DOI:** 10.3390/plants11233227

**Published:** 2022-11-25

**Authors:** Flóra Kolberg, Brigitta Tóth, Deepali Rana, Vitor Arcoverde Cerveira Sterner, Anita Gerényi, Ádám Solti, Imre Szalóki, Gyula Sipos, Ferenc Fodor

**Affiliations:** 1 Department of Plant Physiology and Molecular Plant Biology, ELTE Eötvös Loránd University, 1/C Pázmány P. sétány, H-1117 Budapest, Hungary; 2 Institute of Food Science, Faculty of Agricultural and Food Sciences and Environmental Management, University of Debrecen, 138 Böszörményi út., H-4032 Debrecen, Hungary; 3 Doctoral School of Environmental Sciences, ELTE Eötvös Loránd University, Pázmány Péter lane 1/a, H-1117 Budapest, Hungary; 4 Institute of Nuclear Techniques, Budapest University of Technology and Economics, 9 Műegyetem rkp., H-1111 Budapest, Hungary; 5 Agricultural Research and Development Institute, 30 Szabadság út., H-5540 Szarvas, Hungary

**Keywords:** *Elymus elongatus*, energy grass, Fe-Zn interaction, iron uptake, Szarvasi-1, Zn stress, Zn uptake, *Thinopyrum obtusiflorum*

## Abstract

*Thinopyrum obtusiflorum* (syn. *Elymus elongatus* subsp. *ponticus*) cv. Szarvasi-1 (Poaceae, Triticeae) is a biomass plant with significant tolerance to certain metals. To reveal its accumulation capacity, we investigated its Zn uptake and tolerance in a wide range: 0.2 to 1000 µM Zn concentration. The root and shoot weight, shoot length, shoot water content and stomatal conductance proved to be only sensitive to the highest applied Zn concentrations, whereas the concentration of malondialdehyde increased only at the application of 1 mM Zn in the leaves. Although physiological status proved to be hardy against Zn exposure, shoot Zn content significantly increased in parallel with the applied Zn treatment, reaching the highest Zn concentration at 1.9 mg g^−1^ dry weight. The concentration of K, Mg and P considerably decreased in the shoot at the highest Zn exposures, where that of K and P also correlated with a decrease in water content. Although the majority of microelements remained unaffected, Mn decreased in the root and Fe content had a negative correlation with Zn both in the shoot and root. In turn, the application of excessive EDTA maintained a proper Fe supply for the plants but lowered Zn accumulation both in roots and shoots. Thus, the Fe-Zn competition for Fe chelating phytosiderophores and/or for root uptake transporters fundamentally affects the Zn accumulation properties of Szarvasi-1. Indeed, the considerable Zn tolerance of Szarvasi-1 has a high potential in Zn accumulation.

## 1. Introduction

Plants that grow fast and produce large biomass have a high potential for energy production. Since biomass plants are not for animal forage or human consumption, plantations can be established on degraded or even contaminated lands. Fast growing plants may also efficiently take up contaminating metals so they can also be applied in combined phytoremediation projects [1]. Regarding metal contaminations, zinc accumulation is one of the most important environmental challenges that affect plant cultivation. Uncontaminated soils typically contain Zn in a wide range from 10 to 100 mg kg^−1^. In Europe, there are multiple areas containing more than 100 mg kg^−1^ Zn by geological reasons [2]. As an important industrial pollutant, Zn impacts both terrestrial and aquatic systems, especially on sites of Pb-Zn mining activities like at Guangxi [3] or Hunan [4], in southwest China. Large urban areas such as Budapest, Hungary may also be heavily contaminated with Pb and Zn, primarily at abandoned industrial sites [5].

As an essential metal, zinc plays a role as cofactor in the proteins of catalytic, regulatory and structural roles of plants. Among others, it is a cofactor of carbonic acid anhydrase, Cu/Zn superoxide dismutases and Zn-finger proteins [6]. Indeed, due to environmental factors such as alkaline soil pH, flooding, anoxic conditions, high soil matter and carbonate phosphorus, a large proportion of agricultural lands provide limited Zn availability for the plants. Thus some 17% of the human population is endangered for Zn deficiency due to nutritional backgrounds [7]. On the other hand, Zn may become toxic when available at excessive concentrations for most plants. Toxicity symptoms emerge at concentrations [Zn]_leaf_ > 300 mg Zn kg^−1^ dry matter, although some crops show toxicity symptoms at [Zn]_leaf_ < 100 mg Zn kg^−1^ dry matter [8,9]. This threshold varies in a wide range among taxa and also depends on environmental conditions and on the availability of other heavy metals. In general, Zn excess causes reduced biomass, stunted growth, Fe deficiency-induced chlorosis, interference with phosphorus and inhibition of photosynthetic electron transport; and it has a regulatory influence on stomatal opening. Growth retardation in ryegrass (*Lolium perenne*) occurs at 1–10 mM ZnSO_4_, with a full growth inhibition developing at 50 mM ZnSO_4_ [10]. Zn treatments (75 µM and 150 µM of ZnSO_4_) considerably reduced fresh and dry biomass of below- and above-ground *Sorghum bicolor* plant parts grown in hydroponic culture [11]. Ryegrass exposed to different levels of ZnSO_4_ shows a net decline in the actual and maximal quantum efficiencies of the photosystem II (PSII) reaction centres of the photosynthetic apparatus [10]. Certain taxa are characterised, indeed, to take up Zn in a supraoptimal concentration. Hyperaccumulators may accumulate Zn in the shoot without growth and physiological limitations [12,13,14,15]. Among others, *Noccea caerulescens* (Brassicaceae) may hyperaccumulate Zn in the shoot at concentrations higher than 1% in dry weight [15]. Zinc accumulators can also be found within the grass family (Poaceae/Poales). Several grass species were examined through a hydroponic screening with 100 µM ZnSO_4_ supplementation, and the results showed that oat (*Avena sativa*) and barley (*Hordeum vulgare*) tolerated and accumulated the most Zn. In barley, 2437 µg Zn g^−1^ was observed, whereas oat accumulated 958 µg Zn g^−1^ in shoot biomass [16]. Ryegrass accumulated a maximum of 584 µg Zn g^−1^ in shoot dry matter [17]. *Juncus acutus* (Juncaeae/Poales) was also shown to be hypertolerant to zinc, moreover, concentrations above 500 µg g^−1^ Zn were recorded in its tiller tissues [18].

The uptake in the form of Zn^2+^ is facilitated by the zinc-regulated transporter/iron-regulated transporter-related protein (ZIP) family cation transporters. *Arabidopsis thaliana* ZIP4 is a major component in the regulation of Zn uptake, since it encodes a plasma membrane-localized Zn specific transporter [19]. In rice (*Oryza sativa*) and barley (*Hordeum vulgare*), multiple ZIP family transporters of tissue-specific expression and various Zn regulation were identified [20,21,22], indicating a functional divergence of ZIPs in grasses [23]. Moreover, several grasses have a unique Zn uptake mechanism that is independent from ZIPs. This strategy is based on the secretion of mugineic acid family phytosiderophores (MAs) into the rhizosphere, forming stable complexes mainly with Fe^3+^ ions (the so called Strategy-II of Fe uptake). However MAs are also able to mobilize other elements in the soil, such as Zn; therefore, a competition can occur between these elements for the available metal chelating ligands [24,25]. Barley takes up Zn in the form of Zn deoxymugineic acid (Zn-DMA) complexes by roots [26]. Inside the cell, Zn is bound to ligands, like nicotianamine, oligopeptides, amino acids or organic acids [27]. Heavy metal ATPase 4 (HMA4), a plasma membrane localized protein, plays an important role in transporting Zn from the root to the shoot [28]. Metal tolerance protein 1 (MTP1) works as a Zn^2+^/H^+^ antiporter in the tonoplast; therefore, it is necessary in the hyperaccumulating process [29].

Szarvasi-1 energy grass (*Elymus elongatus* subsp. *ponticus* (Podp.) Dorn; syn. *Thinopyrum obtusiflorum* (DC.) Banfi cv. Szarvasi-1) is an important biomass plant increasingly applied in the agricultural utilization of low quality lands. It is perennial but not invasive, and it can be cultivated as common cereals. The dry matter yield can reach 15 tons per hectare. The biomass is commonly used among others for biogas production and cattle forage. Szarvasi-1 was characterised for performance in different soil types and nutritional supply in terms of biomass production, habitat preference, gas exchange, crop management and weed and fungal infection [30,31]. Uptake of and tolerance against certain heavy metals have already been described under controlled environment in hydroponics [32,33]. Indeed, our knowledge on the tolerance mechanisms of Szarvasi-1 against Zn, a contaminant that has an increasing importance, is still scarce. Here we focus on how Szarvasi-1 is able to maintain the balance between essential transition metals, growth and biomass production under an excess of Zn.

## 2. Results

### 2.1. Plant Growth Parameters

**Figure 1:**
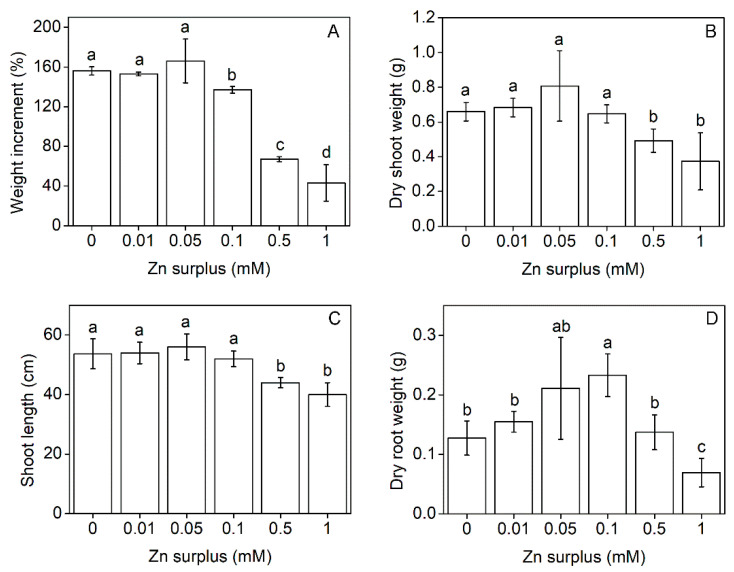
Total fresh biomass accumulation (root + shoot) during the Zn treatment period as a percentage of the initial fresh weight (**A**). Dry matter content in the shoots (**B**), shoot length (**C**) and dry matter content in the roots (**D**) of 50-day-old Szarvasi-1 energy grass grown in nutrient solutions amended with different surplus Zn concentrations for 2 weeks. To compare differences among the treatments, one-way ANOVA was performed with Tukey-Kramer multiple comparisons post hoc test (*p* < 0.05, n = 9), indicated by letters. Error bars represent SD values.

Szarvasi-1 plants were grown in a nutrient solution spiked with a wide range of Zn from 0 to 1 mM above the basic Zn concentration. Zinc concentrations up to 0.05 mM did not affect the total fresh biomass accumulation of plants (root + shoot) compared to the control. Concentrations above that value caused a decrease in the total biomass accumulation under the time of treatment (Figure 1A). The shoot dry weight at harvest reflected the similar effect. Zn up to 0.1 mM concentration had no inhibitory effects on shoot growth, but the plants grown in zinc concentrations above 0.1 mM showed a decrease in the shoot dry weight (Figure 1B). Examining the root dry weight, we discovered that at 0.05 and 0.1 mM Zn concentration, root growth was stimulated compared to the control, but it was significantly inhibited at 1 mM Zn (Figure 1D).

Shoot length changed similarly to shoot dry weight: significantly smaller values were measured only above 0.1 mM Zn concentration (Figure 1C). Despite that, the determined pH value was about 4 in all fresh nutrient solutions amended with different Zn concentrations; after 2 weeks of treatment, the used nutrient solutions reached higher pH values, from 7.8 to 6.2 with increasing Zn concentrations (Table 1).

### 2.2. Physiological Performance of Leaves

**Figure 2:**
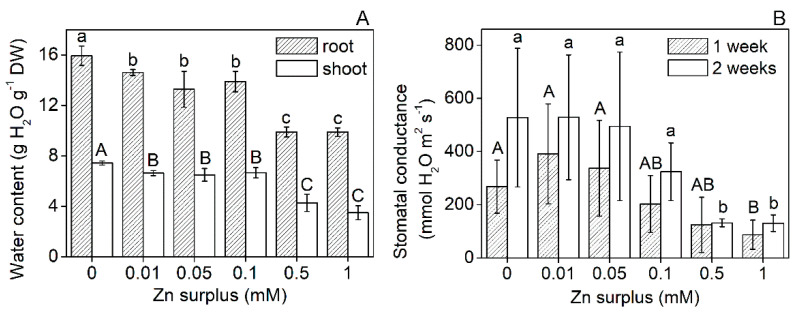
Root and shoot water content after 2 weeks (**A**) and stomatal conductance after 1 and 2 weeks (**B**) of Zn treatment of Szarvasi-1 energy grass grown in nutrient solutions amended with different Zn concentrations. To compare differences among the treatments, one-way ANOVA was performed with Tukey-Kramer multiple comparisons post hoc test (*p* < 0.05, n = 9). Statistically different values are indicated by different letters. Error bars represent SD values.

**Figure 3:**
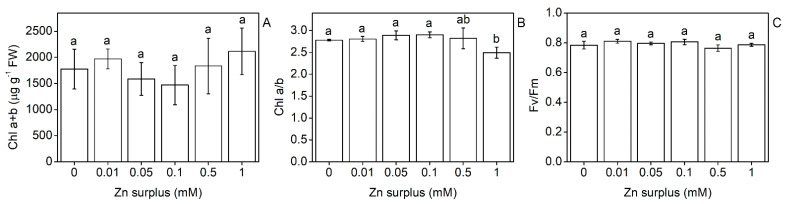
Chlorophyll *a* + *b* concentration (**A**), chlorophyll *a*/*b* ratio (**B**) and the maximal quantum efficiency of PSII in the leaves of 50-day-old Szarvasi-1 energy grass grown in nutrient solutions amended with different surplus Zn concentrations for 2 weeks (**C**). To compare differences among the treatments, one-way ANOVA was performed with Tukey-Kramer multiple comparisons post hoc test (*p* < 0.05, n = 9). Statistically different values are indicated by different letters. Error bars represent SD values.

**Figure 4:**
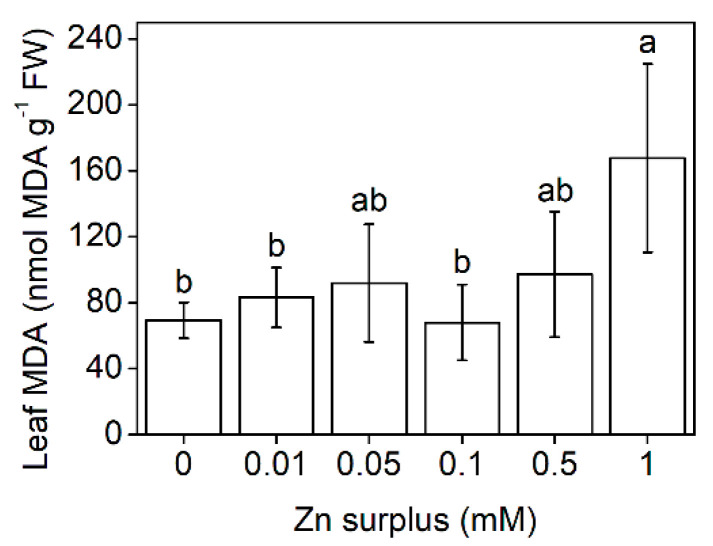
MDA concentration in the leaves of 50-day-old Szarvasi-1 energy grass grown in nutrient solutions amended with different surplus Zn concentrations for 2 weeks. To compare differences among the treatments, one-way ANOVA was performed with Tukey-Kramer multiple comparisons post hoc test (*p* < 0.05, n = 9). Statistically different values are indicated by different letters. Error bars represent SD values.

With increasing Zn concentrations, the water content of the leaves decreased gradually and significantly. At 1 mM zinc, the reduction exceeded 50% compared to the control (Figure 2A). This tendency was also observed in the roots. Transpiration rates of the youngest fully developed leaves measured as stomatal conductance showed similarities with the changes in the water content; however, only the highest Zn concentrations (0.5 and 1 mM Zn) caused a significant decrease (Figure 2B). No significant difference could be defined between the different treatments regarding chlorophyll (Chl) *a* + *b* content, Chl *a*/*b* and the maximal quantum efficiency of PSII except for 1 mM Zn surplus that caused a 10% decrease in Chl *a*/*b* (Figure 3A–C). Oxidative stress caused by elevated Zn concentrations was assessed by measuring the malondialdehyde (MDA) concentration. Compared to the control, only 1 mM Zn resulted in increased MDA concentration in the leaves (Figure 4).

### 2.3. Element Composition of the Shoots

**Figure 5:**
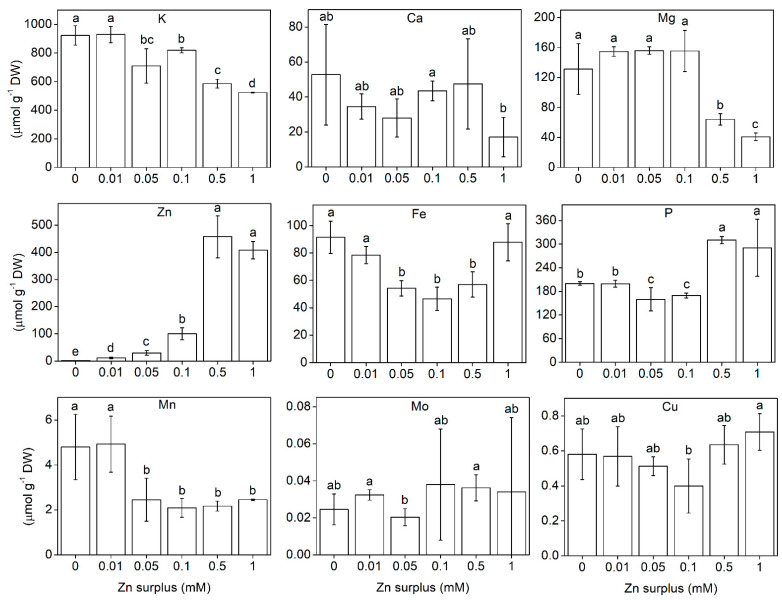
K, Ca, Mg, P, Zn, Fe, Mn, Mo and Cu concentration in the roots of 50-day-old Szarvasi-1 energy grass grown in nutrient solutions amended with different surplus Zn concentrations for 2 weeks. The measurements were made by ICP-MS. To compare differences among the treatments, one-way ANOVA was performed with Tukey-Kramer multiple comparisons post hoc test (*p* < 0.05, n = 9). Statistically different values are indicated by different letters. Error bars represent SD values.

**Figure 6:**
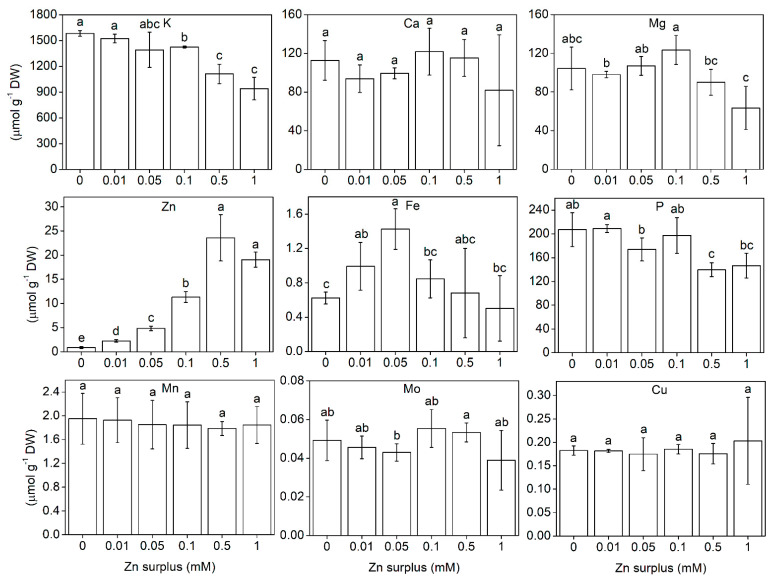
K, Ca, Mg, P, Zn, Fe, Mn, Mo and Cu concentration in the shoots of 50-day-old Szarvasi-1 energy grass grown in nutrient solutions amended with different surplus Zn concentrations for 2 weeks. The measurements were made by ICP-MS. To compare differences among the treatments, one-way ANOVA was performed with Tukey-Kramer multiple comparisons post hoc test (*p* < 0.05, n = 9). Statistically different values are indicated by different letters. Error bars represent SD values.

Element concentration was determined from whole root and shoot samples. The element concentration in the roots can be seen in Figure 5. K accumulation showed significant decrease with increasing Zn surplus concentrations. Interestingly, Mg significantly decreased, while P significantly increased at 0.5 and 1 mM Zn treatment. Ca was not affected by Zn treatments. Fe concentration was significantly lower at 0.05, 0.1 and 0.5 mM Zn than at lower and higher Zn surplus concentrations. Mo and Cu did not change but Mn was reduced significantly at 0.05 mM Zn surplus concentration, then it remained the same. In the shoot, the concentration of K also considerably decreased at the two highest Zn concentrations applied (Figure 6). This was in correlation with the changes in the physiological parameters such as water content. Mg and P also showed a decreasing tendency, but the change was not significant. Ca, Mn, Mo and Cu did not show changes over the Zn concentration range in the nutrient solution. The change in Fe concentration was different from the other elements. It changed inversely compared to the shoot: significantly increased up to 0.05 mM Zn treatment then it started to decrease, though the latter values were not significantly different from the control.

Zinc concentrations in the roots and leaves increased with increasing Zn concentration in the nutrient solutions. Zinc accumulation was very significant above 0.01 mM Zn treatments compared to the control. The highest shoot Zn concentration in a single measurement was 28.9 μmol g^−1^ dry mass i.e., 1.9 mg g^−1^ dry mass Zn at the 0.5 mM treatment, whereas the mean was 23.6 μmol Zn g^−1^ dry mass (Figure 6).

Concerning the pattern of Zn localisation in the different parts of the leaves, Zn accumulated in the young, developing leaves of the plants treated with 0.5 mM Zn. Older, more developed leaves showed lower Zn levels. There was no significant difference in the Zn intensities between the basal, middle and apical sections of the leaves (Supplementary Figure S1).

### 2.4. Effect of Iron Supply on Zinc Accumulation

**Figure 7:**
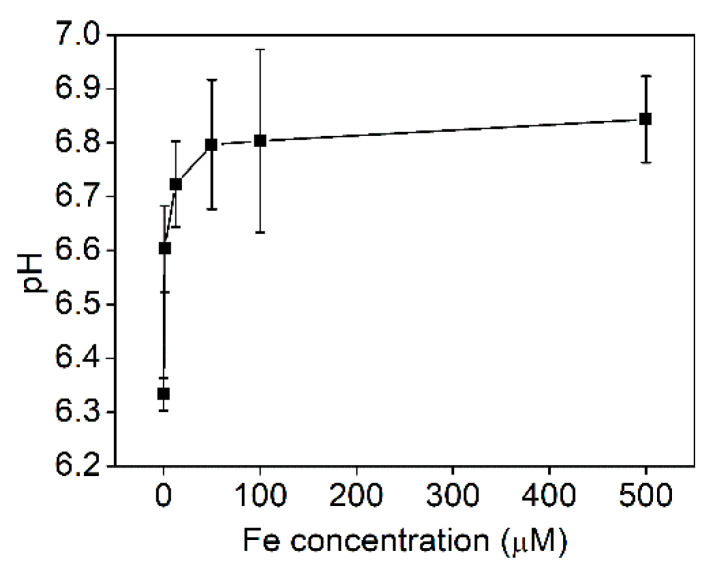
pH of the nutrient solution of Szarvasi-1 energy grass pre-grown for two weeks in nutrient solution containing 0–500 µM Fe and then spiked with 100 µM Zn for an additional week (mean ± SD, n = 3).

**Figure 8:**
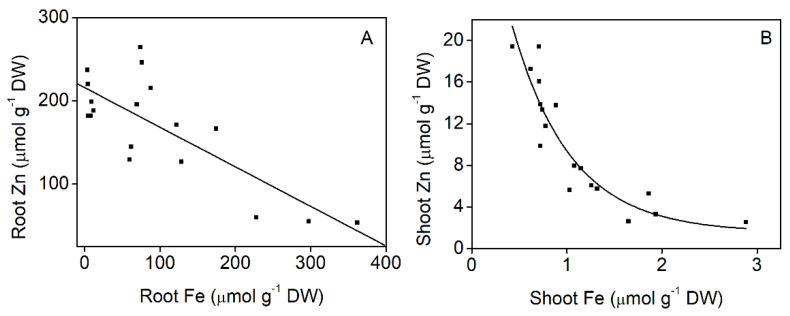
Correlation of Zn and Fe concentration in the roots (**A**) and shoots (**B**) of Szarvasi-1 energy grass pre-grown for two weeks in nutrient solution containing 0–500 µM Fe and then spiked with 100 µM Zn for an additional week (data of individual plant samples are plotted). ((**A**) Linear fit: R^2^ = −0.7849, *p* < 0.0001; (**B**) Curve fit: Chi^2^ = 5.3288, R^2^ = 0.8544).

In order to reveal the connection between Fe and Zn uptakes, we conducted separate, shorter experiments, in which we applied a wide range of Fe concentrations in the nutrient solution, while the Zn treatment was the same: 0.1 mM. The pH of the nutrient solution at harvest was in the slightly acidic range in contrast to that of older plants. It sharply increased from 6.3 to 6.8, where it reached a saturation point at 0.05 mM Fe (Figure 7). The shoot Fe concentration continuously increased with the increasing Fe treatment, which in turn resulted in continuous decrease in the Zn accumulation. The highest Zn accumulation was measured in the plants with total Fe deprivation during the (Fe and Zn) treatments (Supplementary Figure S2A). The same tendency was observed in the root Fe and Zn concentration (Supplementary Figure S2B). The negative correlation between root and shoot Fe and Zn concentration is represented in Figure 8A,B. The ferric chelate reductase (FCR) activity of the root tips was not measurable up to 0.0125 mM Fe supply, and it did not exceed 4 nmol Fe g^−1^ min^−1^ at higher external Fe supply either (Supplementary Figure S3).

### 2.5. Effect of EDTA on Zinc and Iron Accumulation and Interaction

As EDTA is a strong chelator for both Fe and Zn, it was applied in excessive concentrations to further analyse the interaction between Zn and Fe uptake (Table 2). Zn accumulation increased in both roots and shoots with increasing surplus Zn concentration similarly to the Fe-citrate-grown plants, while Fe concentration did not change significantly. A large surplus of EDTA significantly lowered Zn and Fe concentration in the roots. Zn in the shoot showed a decrease with increasing surplus EDTA, which became significant at the highest concentration applied in this experiment. Although changes in Fe concentration in the shoots were not significant, 0.5 mM EDTA seemed to stimulate Fe translocation at 0 and 0.1 mM surplus Zn treatment.

## 3. Discussion

### 3.1. Growth and Physiological Responses of Szarvasi-1 to Surplus Zinc

Szarvasi-1 increased the pH in the acidic nutrient solution in the experimental setup, in accordance with its preference to alkaline soil pH between 6.5 and 10 [30]. Surplus Zn in the nutrient solution, up to 0.1 mM, also resulted in an increase in the pH to 7.6–7.8 (Table 1). Alteration of the pH of the nutrient solution refers to the high nitrate utilization which is normally coupled with OH^−^ or HCO_3_^−^ efflux of roots [9]. However, massive Zn excess (0.5 and 1.0 mM) decreased the alkalinisation effect, and, thus, the nutrient solution remained in the acidic range. Similarly to the effects on the nutrient medium, physiological parameters of the plants such as biomass and growth parameters were considerably affected also by the two highest Zn concentrations only (Figure 1). In contrast, a moderate excess of Zn (0.05 and 0.1 mM) resulted in eustress conditions with an insignificant shoot but significant root growth stimulation, in agreement with our previous work [33] and data obtained on ryegrass [17] and barley [34]. Growth of Zn hyperaccumulators is enhanced by high Zn concentration. Tolrà et al. [35] found that biomass of *Noccea caerulescens* is stimulated up to 0.5 mM Zn concentration. In contrast, shoot biomass of barley and oat was reduced by 30 and 20%, respectively, compared to control at 0.1 mM Zn treatment. Moreover, dicots such as Indian mustard (*Brassica juncea*) may suffer above 80% shoot biomass reduction upon Zn exposure [16]. Since the shoot dry mass of Szarvasi-1 was unaffected by medium Zn exposure, and 1 mM Zn resulted in only a 50% reduction of biomass production, the taxon performs an efficient Zn tolerance.

The water relations showed sensitivity to the Zn surplus only at the two highest concentrations (Figure 2A). Above 0.1 mM Zn concentration, Zn stress markedly decreased stomatal conductance (Figure 2B). On sugar beet (*Beta vulgaris*), Sagardoy et al. [36] indicated that 0.3 mM Zn treatment resulted in a 70% loss of stomatal conductance and decreased relative water content of leaves together with morphological changes in stomata and mesophyll tissues. Although Zn does not change its valence in biological environments, Zn exposure results in the formation of reactive oxygen species (ROS) [37,38], causing lipid peroxidation and increased membrane permeability. The concentration of membrane damage indicator MDA increased significantly only at 1 mM Zn (Figure 4), where the proposed membrane leakage could have contributed to the lowered shoot relative water content. Over the water regime of leaves, Zn excess can also alter the operation of the photosynthetic apparatus, resulting, e.g., in decreased electron transport rate [39]. In comparison, neither structural nor functional parameters of the photosynthetic apparatus were altered in Szarvasi-1 by Zn exposure except for a minor decrease in Chl *a*/*b* at 1 mM surplus Zn (Figure 3). Similarly, the photosynthetic apparatus of sugar beet was also proved to be quite insensitive against Zn stress [36,40].

### 3.2. Zn Accumulation

In previous studies, applying Cd, Cu, Ni, Pb and Zn treatment to the nutrient solution at 0.01 mM concentration, Szarvasi-1 was found to be sensitive to Cd and Cu but tolerant to Ni and Pb [32,33]. Sipos et al. [33] have reported that 336 μg g^−1^ Zn in the shoot of Szarvasi-1 energy grass grown in nutrient solution containing 0.01 mM Zn induced growth stimulation due to slightly increased Fe translocation and possible efficient detoxification mechanisms. In our present study, shoot Zn concentration increased together with the degree of Zn excess. Accumulation of Zn in the tissues reached a maximum of 23.6 μmol g^−1^ which is 1543 μg g^−1^ Zn at 0.5 mM Zn exposure (Figure 6), about half the Zn content of hyperaccumulators grown in soil. In the dried aboveground plant parts, a threshold of 10,000 μg g^−1^ Zn for Zn hyperaccumulation was first stated [12], and that threshold is exceeded by *A. halleri* and *N. caerulescens*. However, in more recent works, a 3000 μg g^−1^ threshold value for Zn was suggested [13,14,15]. In comparison, sugar beet accumulates 260 μg g^−1^ Zn in dry matter at 0.1 mM Zn treatment [40]. Wheat is able to accumulate 1124 μg g^−1^ Zn in the leaves at 3 mM Zn treatment [41]. A comparative study, including several grass species for Zn removal efficiency from nutrient solution containing 0.1 mM Zn, indicated a decreasing Zn accumulation in the following sequence: *Hordeum vulgare* > *Avena sativa* > *Elymus junceus* > *Elymus elongatus*, etc. [16]. In the same study a 50% growth reduction was reported for *E. elongatus*, whereas, in the present study, Szarvasi-1 showed no growth retardation by the same Zn concentration. Furthermore, *H. vulgare* and *A. sativa* accumulated about 700 and 500 μg g^−1^ Zn along with 30 and 15% shoot growth reduction, respectively, while Szarvasi-1 accumulated over 740 μg g^−1^ Zn in dry shoot biomass. Based on these results, Szarvasi-1 is a good candidate for Zn removal from contaminated substrates, i.e., for phytoremediation or recultivation purposes, and should be tested in soil cultures spiked with Zn. Accumulation of Zn is prevalent in young aerial tissues in hyperaccumulators [42]. In Szarvasi-1, we also demonstrated higher Zn accumulation in the young developing leaves compared to the older ones, the growth of which has already terminated (Supplementary Figure S1).

### 3.3. Ionomic Interactions at Zn Exposure

The uptake/accumulation pattern of K, Ca, Mg, P, Mn, Mo and Cu of Szarvasi-1 energy grass did not reveal any specific interaction with Zn (Figure 5 and Figure 6). The decrease in shoot K, Mg and P levels at the highest Zn surplus may be a result of oxidative damages, and, thus, impaired cellular function. K and Mg also decreased in the roots also indicating limitations at the uptake. Similar results were achieved by Brune et al. [34] for K and P but not for Mg. In our study, the P content of the roots increased indicating a significant P retention. The negative interaction between P and Zn is long known [43,44,45]. Although P and Zn are antagonistic in some of their aspects at soil availability [46], retention of P in the roots upon Zn exposure, and, thus, enhanced root-to-shoot translocation of Zn, suggests an altered mobility of phosphates in the xylem sap and might be connected to protective mechanisms against high Zn translocation activity [47]. The microelement levels did not change significantly under the Zn surplus concentration range applied in our study, except for Mn in the roots and Fe in both roots and shoots. In ryegrass, the root Mn concentration was found to decrease, while leaf Mn concentration remained unchanged at 1 mM Zn treatment compared to the control [48]. In Szarvasi-1, we found the same. The drop in root Mn concentration at 0.05 mM Zn surplus concentration may refer to a competition with Zn for root binding sites and uptake, but, as the shoot Mn concentration remained unchanged at all Zn levels, it seems rather unspecific, unlike the interaction between Fe and Zn.

### 3.4. Zinc and Iron Interaction

The interaction of Zn with Fe homeostasis had a dual nature in Szarvasi-1. Root Fe concentration and its alteration under different Zn supplies appeared to be reciprocal to that in the shoot (Figure 5 and Figure 6). Since Szarvasi-1 is a member of the *Hordeinae subtribus* (Poaceae/Pooideae/Hordeeae), close relative to Strategy II Fe uptake model *Hordeum* species, it is suggested to perform a chelation-based root Fe uptake. *Hordeum vulgare* is known to excrete 2′-deoxymugineic acid, mugineic acid and 3-epihydroxymugineic acid [49]. The synthesis and release of these compounds are upregulated under Fe limitation [50]. MAs released by the roots of Strategy II plants for the uptake of Fe also have a high affinity to other metal ions such as Zn. Although Zn and Fe homeostasis can directly interact with each other [26], Fe uptake and translocation is also affected by the P homeostasis. Since the expression of P and Fe deficiency response elements are connected with a reciprocal interaction [51,52], accumulation of P in the roots could have a negative impact on Fe uptake and translocation to the shoot as well.

In order to further elucidate the question, we have conducted a separate experiment with Szarvasi-1 grown in a wide concentration range of Fe in the nutrient solution. Zinc was applied in 0.1 mM concentration at all Fe concentrations for a one week period, and then the shoots and roots were analysed for Zn and Fe content. We have found a negative correlation between the two metals: as the Fe was increased in the solution, Fe increased but Zn decreased in both roots and shoots (Figure 8, Supplementary Figure S2). In rice, the signalling of the presence of Fe and Zn in the tissues is based on OsHRZ and OsIDEF1 that regulates the Fe uptake-related genes [53]. High Fe (and low Zn) concentration in roots and in the stele induces both OsIDEF1 and OsHRZ that suppress the iron uptake machinery, whereas low Fe (and high Zn) concentration supresses OsHRZ that leads to the expression of the Fe uptake machinery and iron translocation towards to shoots. We also suppose that Fe and Zn may be in competition for the chelating ligands released by plant roots to the nutrient solution. However, it cannot be excluded that in the young energy grass plants, a ferrous Fe uptake transporter may also be present. *A. thaliana* iron regulated transporter 1 (IRT1) may transport both Fe and Zn to the root cells [54,55]. Orthologues of AtIRT1 generally express in roots of Strategy I plants, participating in the transport of both Fe and Zn. Vatansever et al. [56] identified the orthologues of IRT1 in *Brachypodium distachyon*, *Sorghum bicolor*, *Zea mays* and *Oryza sativa* that makes its general existence in Triticinae very likely as well. In *Hordeum vulgare*, 2 µM Zn increased, whereas a few days long exposure to 1000 µM Zn decreased the expression of *HvIRT1* [57]. In *A. thaliana*, excess Zn in the roots also causes a decrease in the expression of *IRT1* [58]. Although the cellular mechanism of Zn sensing and signalling has not been completely revealed so far, overlapping between Fe and Zn signalling has also been proposed [51]. With the lack of transcriptomic data, the expression of an IRT1 orthologue in Szarvasi-1 cannot be assured, but its close relation to *Hordeum* spp. makes it very likely and would provide another site of competition for the two metals. On the other hand, the application of Fe-EDTA instead of Fe-citrate reveals that the apoplastic binding of Fe and Zn is largely controlled by available chelating agents and the stability of their complexes (Table 2). Surplus EDTA ensures the proper solubility and availability of Fe at all times, but may also enhance the root-to-shoot Fe translocation. In the meantime, root Zn uptake and its translocation decreases, indicating that free Zn^2+^ ions may at least partly contribute to uptake and Fe availability at Zn exposure is also a limiting factor.

FCR activity of young Szarvasi-1 plants was low and not inducible by Fe deficiency as it is in most Strategy I plants, but the H^+^ efflux increased with decreasing Fe (Supplementary Figure S3, Figure 7). This may refer to the high H^+^ demand of Fe-phytosiderophore symporters and that the roots do not reduce Fe in Szarvasi-1. FCR activity is related to ferric reductase oxidase (FRO) family enzymes found in the plasma membrane of root cells of non-graminaceous plants and is responsible for the reduction of ferric Fe as a prerequisite for Fe uptake. In *A. thaliana*, expression of *FRO2* is especially increased in the root tips under Fe deficiency along with *IRT1*. In *Oryza sativa* and *Zea mays*, which are both Strategy II plants, phytosiderophores are also released for Fe uptake, and the expression of *IRT1* orthologues was reported as well [55]. This implies the co-existence of the different transport systems where Strategy II may increase their efficiency to acquire nutrients from the soil, but certain components of the underlying Strategy I may also perform as a platform of elemental interactions.

## 4. Materials and Methods

### 4.1. Plant Material and Treatments

Szarvasi-1 (*Elymus elongatus* subsp. *ponticus* (Podp.) Melderis cv. Szarvasi-1) seeds were germinated on wet filter paper in Petri dishes at room temperature and sunlight for one week. Three seedlings with about 5–6 cm shoots were placed on a 2 cm wide strip of sponge-rubber, rolled up and fastened in a hole (35 mm in diameter) cut in a polystyrene plate. The plates with 9 groups of seedlings were placed to plastic buckets filled up with 5 L quarter-strength Hoagland nutrient solution of the following composition: 1.25 mM KNO_3_; 1.25 mM Ca(NO_3_)_2_; 0.5 mM MgSO_4_; 0.25 mM KH_2_PO_4,_ 11.6µM H_3_BO_3_; 4.5 µM MnCl_2_·4H_2_O; 0.19 µM ZnSO_4_·7H_2_O; 0.12 µM Na_2_MoO_4_·2H_2_O; 0.08 µM CuSO_4_·5H_2_O and 25 µM Fe(III)-citrate-hydrate (basic nutrient solution). The nutrient solution was continuously aerated and replaced with fresh solution once a week. After 2 weeks, the volume of the solution in each bucket was increased to 10 L. After 4 weeks of pre-cultivation in total, the groups of 3 plantlets still wrapped together in the rubber-sponge were transferred to polystyrene rings of 8 cm in diameter. The rings, each with 3 plantlets, were placed into 0.8 L pots filled up with the same nutrient solution but supplied with different Zn concentrations. In this treatment solution, zinc was added as surplus ZnSO_4_·7H_2_O in 0 (control), 0.01, 0.05, 0.1, 0.5 and 1 mM concentrations above the 0.19 µM Zn already applied in the basic solution. The plants were grown in a climate controlled growth chamber at 20/25 °C, at 75% relative humidity and 150 µmol m^−2^ s^−1^ photosynthetic photon flux density (PPFD) with a 10/14 h dark/light period. Each group contained 3 parallel pots with 3 plants grown together (18 pots in one experiment in total). Physiological parameters were measured and the plants were harvested 14 days after treatment (50-day-old stage) to gain fresh and dry mass and perform analytical measurements.

In separate experiments to study the interaction of Fe and Zn in shoot accumulation, we have grown plants with modifications: (i) the Fe source was replaced in the basic nutrient solution with 25 µM Fe(III)-EDTA, while in the treatment solution surplus ZnSO_4_·7H_2_O was applied in 0, 0.01 and 0.1 mM concentrations complemented with 0, 0.1 and 0.5 mM Na_2_EDTA. The shoot Fe and Zn concentrations were measured after two weeks. (ii) In the basic nutrient solution, the Fe(III)-citrate-hydrate was applied in 0, 1, 12.5, 50, 100 and 500 μM concentrations for two weeks only. Then, the used nutrient solutions were replaced with similar fresh solutions but spiked with 100 μM ZnSO_4_·7H_2_O. The Zn treatment was applied for 7 days when the excised root tips were subjected to FCR test or harvested for analytical measurements (at the 28-day-old stage). The experiment was conducted with 3 parallel pots, each containing 5 plants grown together.

### 4.2. Mass Measurements

Total fresh mass of the whole intact plants was measured before the zinc treatment and at harvest to calculate the increment. Then, at harvest, shoots and roots were separated and weighed (root fresh mass was determined after being centrifuged at 300× *g* between sheets of filter paper for 1 min to remove traces of nutrient solution). The dry mass of all tissues (root and shoot) was determined after drying at 80 °C until a constant weight was achieved. The water content of the roots and shoots was calculated as follows:

(fresh mass − dry mass)/dry mass.

### 4.3. Chlorophyll Concentration

Chl concentration was measured in the middle sections of the youngest fully developed leaves. The concentration of Chl *a* and *b* was determined spectrophotometrically (UV-2101PC, Shimadzu, Japan) from 80% acetone extracts using the equations of Porra et al. [59].

### 4.4. Stomatal Conductance

Stomatal conductance was measured with an AP4 porometer (Delta-T Devices, Cambridge, UK) on the adaxial epidermis of the middle sections of the youngest fully developed leaves. Conductance was calculated as mmol H_2_O m^−2^ s^−1^.

### 4.5. Malondialdehyde Concentration

As a response to various environmental stresses, reactive oxygen species are formed, causing lipid peroxidation of polyunsaturated fatty acids [60]. To determine the extent of lipid peroxidation, we measured the MDA concentration photometrically based on the reaction with thiobarbituric acid [61,62]. A sample of 100 mg of leaf or root tissues was homogenised with 1.25 mL, 0.1% (*v*/*v*) trichloracetic. After centrifuging (1500× *g*, 10 min), 1 mL 20% (*w*/*v*) trichloracetic acid and 1% (*w*/*v*) thiobarbituric acid (4,6-dihydroxi-2-merkaptopyrimidine) were added to the supernatant, and the reaction medium was incubated for 30 min at 90 °C on a shaker. After the reactions the solutions were cooled to room temperature and the absorbance was measured at 532 nm with a Shimadzu UV-2101PC UV-VIS scanning spectrophotometer. MDA content was determined using the coefficient ε = 155 mM^−1^ cm^−1^.

### 4.6. Chlorophyll a Fluorescence Induction

Chl *a* fluorescence induction on leaf samples was performed using a FMM Chl *a* fluorometer (Budapest University of Technology and Economics, Department of Atomic Physics, Budapest, Hungary) [63]. The internal light source is a 635 nm laser diode (QL63H5SA, Roithner Lasertechnik GmbH, Wien, Austria) with 20 mW maximum optical power. Kautsky kinetics were monitored following a 5 min dark adaptation under 5 min excitation time at 690 nm and 735 nm by selective filters, where the minimal (F_0′_), maximal (F_m_) and steady-state (F_s_) fluorescence values were recognized at both wavelengths. The difference between F_0′_ and F_m_ is the variable fluorescence (F_v_). Maximal quantum efficiency of the photosystem II (PSII) reaction centres was calculated as F_v_/F_m_ = (F_m_ − F_0′_)/F_m_ at both emission maxima. Red to far-red Chl *a* fluorescence ratios (F690/F735) were calculated from F_s_ values.

### 4.7. Element Concentration Analysis

Measurements of the dried plant samples were made after acidic digestion. Root samples were thoroughly washed with deionized water to remove traces of nutrient solution. 5–10 mL cc. For overnight incubation, HNO_3_ was added to each gram of the samples. Then the samples were pre-digested for 30 min at 60 °C. After this, 2–3 mL 30% (*m*/*m*) H_2_O_2_ was added for a 90 min boiling at 120 °C. The solutions were filled up to 10–50 mL, homogenised and filtered through MN 640 W filter paper. The element content of the filtrate was determined by ICP-MS. All samples were prepared in triplicate.

### 4.8. Zn Distribution

Preliminary analysis was performed with a 3D XRF spectrometer to estimate the detection limit of XRF analysis on this type of sample [64]. The distribution of Zn in the sample was analysed by a benchtop microXRF analytical instrument (XGT-7200V, Horiba, Japan). The instrument is equipped with a Rh X-ray tube and a silicon drift detector (SDD). The measuring parameters were as follows: (i) irradiated spot diameter on the surface of the target object was 100 µm, (ii) the X-ray tube was operated at high voltage 50 kV and current 1 mA, (iii) for determination of the Zn content the intensity of Zn-Kα (E = 8.637 keV) was measured and (iv) the X-ray tube operated in vacuum chamber; while the sample was placed under atmospheric conditions, the excitation X-ray beam was emitted through a thin polymer window to the sample surface in order to excite its atoms, and one portion of the characteristic Kα and Kβ photons emitted by the Zn atoms was detected by the SDD. The leaves were cut from the plants and immediately fastened on the sample stage and measured.

### 4.9. Ferric Chelate Reductase Assay

FCR activity of root tips was determined as described in Kovács et al. [65]. Excised root tips (2–3 cm long, 20–30 mg) were rinsed with 0.5 mM CaSO_4_ and transferred to a solution containing 500 µM Fe-EDTA, 400 µM BPDS, 2.5 mM KNO_3_, 2.5 mM Ca(NO_3_)_2_, 1 mM MgSO_4_ and 0.5 mM KH_2_PO_4_. The solution was buffered with 5 mM MES (pH = 6) continuously shaken at 100 rpm for 15 min in darkness. Absorbance at 535 nm was determined spectrophotometrically after one hour incubation in dark. The root tips were weighed and the FCR activity was calculated using the specific extinction coefficient of Fe(II)-BPDS_3_^4−^ (22.14 mM^−1^ cm^−1^). The pH of the nutrient solution in which the plants were growing was also determined to quantify H^+^ efflux. Nine parallel samples were used for each measurement.

### 4.10. Statistical Treatment

The experiment was performed three times. Error values represent standard deviations (SD) in figures and tables. For weight and element content measurements, whole shoot and root samples of plants grown together in each pot were used. The significance of difference between data was calculated with one-way analysis of variance (ANOVA) followed by Tukey-Kramer multiple comparisons post hoc test using InStat 3.0 (GraphPad Software, San Diego, CA, USA) software. Statistically different groups (*p* < 0.05) are indicated by lowercase letters.

## 5. Conclusions

Szarvasi-1 energy grass is tolerant against increasing high Zn concentrations in hydroponic solutions. The growth of plants decreased only at the highest Zn levels. Zn uptake and accumulation increased, approaching a level typical to that of hyperaccumulator plants. This may lead to fairly high biomass yield with elevated Zn concentration, but this should be confirmed in soil culture experiments. At increasing surplus Zn concentrations, we found significant decreases in K and Mn concentration in the root, whereas, in the shoot, K, Mg and P also decreased. These changes might be associated with a mild oxidative stress-induced membrane leakage indicated by increased shoot MDA. The Zn accumulation is stimulated by low Fe concentrations, which refer to a competitive uptake mechanism of the two metals in the Triticinae plant Szarvasi-1. However, excessive EDTA application, which maintains a proper Fe supply, lowers Zn accumulation in roots and shoots. The Fe-Zn competition for Fe chelating phytosiderophores and/or for root uptake transporters fundamentally affects the Zn accumulation properties of Szarvasi-1. Based on our results, Szarvasi-1 can be recommended to apply in the recultivation or phytoremediation of degraded, high Zn, alkaline soils with poor Fe availability. Produced biomass may be applied only for non-forage purposes.

## Figures and Tables

**Table 1:** pH of the treatment nutrient solution of 50-day-old Szarvasi-1 energy grass amended with different surplus Zn concentrations for 2 weeks. To compare differences among the treatments, one-way ANOVA was performed with Tukey-Kramer multiple comparisons post hoc test (*p* < 0.05, mean ± SD, n = 3). Significantly different values are indicated by different letters.

Surplus Zn (mM)	Initial pH ^1^	pH at Harvest
0	4.07 ± 0.05 e	7.82 ± 0.09 a
0.01	3.99 ± 0.04 e	7.84 ± 0.02 a
0.05	4.05 ± 0.05 e	7.66 ± 0.19 ab
0.10	4.01 ± 0.04 e	7.63 ± 0.09 b
0.50	4.03 ± 0.06 e	6.71 ± 0.04 c
1.00	3.99 ± 0.04 e	6.21 ± 0.26 d

^1^ pH of the stock treatment solutions.

**Table 2:** Zn and Fe concentration in the roots and leaves of 50-day-old Szarvasi-1 energy grass in nutrient solutions amended with different surplus Zn and Na_2_EDTA concentrations for 2 weeks. The measurements were made by ICP-MS. To compare differences among the treatments, one-way ANOVA was performed with Tukey-Kramer multiple comparisons post hoc test (*p* < 0.05, n = 9). Statistically different values are indicated by different letters. Error bars represent SD values.

Treatments		Roots		Leaves	
Surplus Zn (mM)	Na_2_EDTA (mM)	Zn (µmol g^−1^)	Fe (µmol g^−1^)	Zn (µmol g^−1^)	Fe (µmol g^−1^)
0	0	1.13 ± 0.09 d	55.02 ± 11.81 a	0.62 ± 0.11 d	0.73 ± 0.24 a
0	0.1	0.46 ± 0.16 d	2.60 ± 0.62 c	0.39 ± 0.02 d	0.74 ± 0.25 a
0	0.5	0.63 ± 0.12 d	1.36 ± 0.37 c	0.43 ± 0.09 d	1.45 ± 0.53 a
0.01	0	58.57 ± 6.79 b	59.30 ± 16.64 a	5.52 ± 0.38 c	0.66 ± 0.30 a
0.01	0.1	11.98 ± 1.40 c	3.06 ± 0.12 c	3.46 ± 0.57 c	0.81 ± 0.14 a
0.01	0.5	9.47 ± 3.41 c	1.12 ± 0.91 c	3.29 ± 0.62 c	0.54 ± 0.21 a
0.10	0	178.57 ± 15.27 a	59.70 ± 10.07 a	25.96 ± 3.98 a	0.48 ± 0.09 a
0.10	0.1	137.96 ± 19.06 a	20.16 ± 4.01 b	15.88 ± 3.00 b	0.73 ± 0.19 a
0.10	0.5	33.42 ± 7.54 b	0.82 ± 0.16 c	16.69 ± 1.01 b	1.32 ± 0.64 a

## Data Availability

The data presented in this study are available on reasonable request from the corresponding author.

## Supplementary Materials

The following supporting information can be downloaded at https://www.mdpi.com/article/10.3390/plants11233227/s1, Figure S1: Localization of Zn in the young (developing) and old leaves of 50-day-old Szarvasi-1 energy grass grown in nutrient solution amended with 0.5 mM Zn for 2 weeks; Figure S2: Fe and Zn concentration in the shoots (A) and roots (B) of 28-day-old Szarvasi-1 energy grass pre-grown for two weeks in nutrient solution containing 0–500 µM Fe then spiked with 100 µM Zn for an additional week; Figure S3: Ferric chelate reductase activity of the root tips of 28-day-old Szarvasi-1 energy grass pre-grown for two weeks in nutrient solution containing 0–500 µM Fe then spiked with 100 µM Zn for an additional week.
