# Supplementary material for: Iron Status Affects the Zinc Accumulation in the Biomass Plant Szarvasi-1
Source: Plants (Basel). 2022 Nov 25;11(23):3227. doi: 10.3390/plants11233227 (PMC9738582; doi:10.3390/plants11233227)
Supplement: Supplementary file 1 [file plants-11-03227-s001.zip › plants-2004837-supplementary.pdf]

Iron status affects the Zinc accumulation in the biomass plant Szarvasi-1

Flóra Kolberg, Brigitta Tóth, Deepali Rana, Vitor Arcoverde Cerveira Sterner, Anita Gerényi, Ádám Solti, Imre Szalóki, Gyula Sipos and Ferenc Fodor

Supplementary figures:

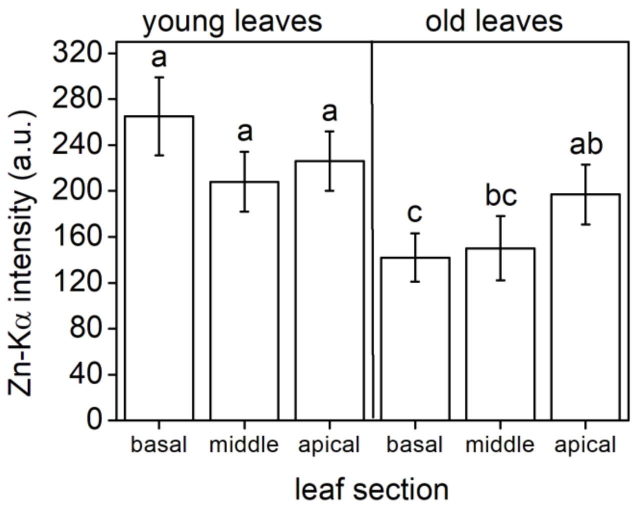

Supplementary Figure S1. Localization of Zn in the young (developing) and old leaves of 50-day-old Szarvasi-1 energy grass grown in nutrient solution amended with 0.5 mM Zn for 2 weeks. The measurements were made by a microXRF instrument (Horiba, Japan). To compare differences among the treatments, one-way ANOVA was performed with Tukey-Kramer multiple comparisons *post hoc* test ($p < 0.05$, n = 3). Statistically different values are indicated by different letters. Error bars represent SD values.

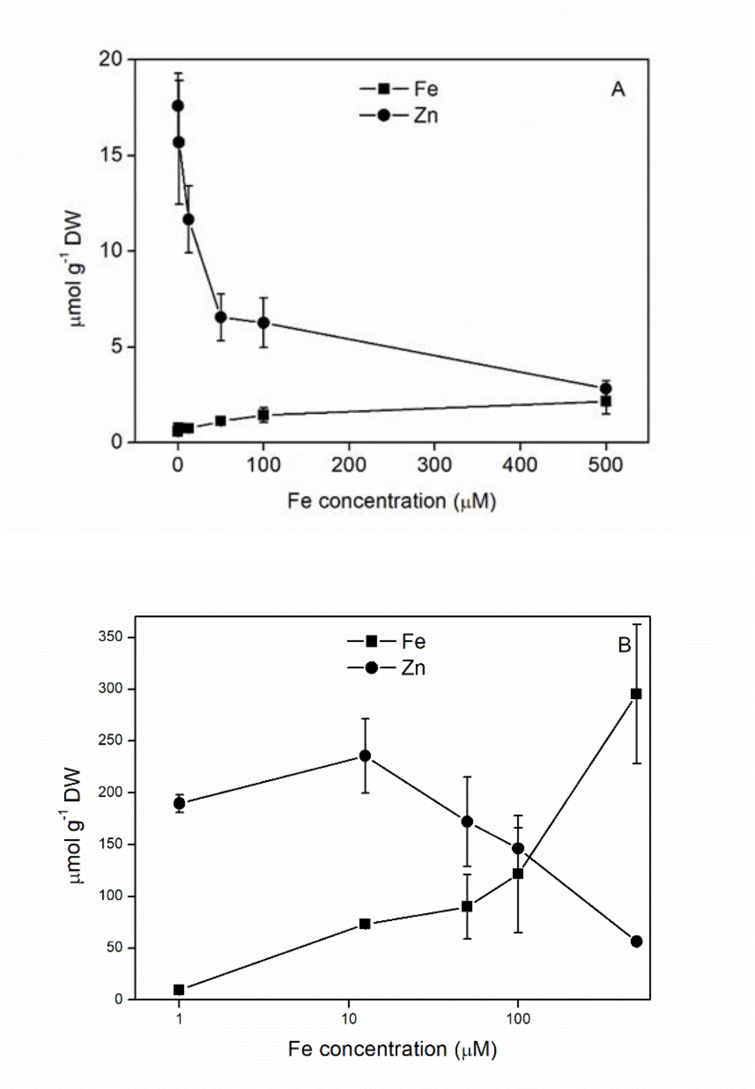

Supplementary Figure S2. Fe and Zn concentration in the shoots (A) and roots (B) of 28-day-old Szarvasi-1 energy grass pre-grown for two weeks in nutrient solution containing 0-500 μM Fe then spiked with 100 μM Zn for an additional week (n = 3, mean ± SD).

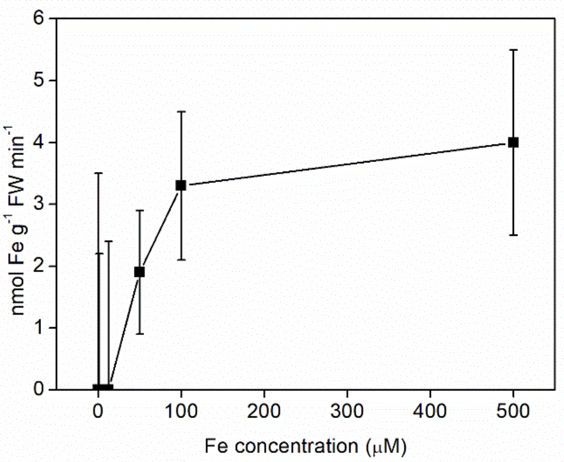

Supplementary Figure S3. Ferric chelate reductase activity of the root tips of 28-day-old Szarvasi-1 energy grass pre-grown for two weeks in nutrient solution containing 0-500 μM Fe then spiked with 100 μM Zn for an additional week (n = 3, mean ± SD).
